# Biomarkers Use and Development in Hepatology: Insights on the Latest Applications

**DOI:** 10.3390/cells12010104

**Published:** 2022-12-27

**Authors:** Alessandra Mangia

**Affiliations:** Liver Unit, Department of Medical Sciences, Fondazione “Casa Sollievo della Sofferenza” IRCCS, 71013 San Giovanni Rotondo, Italy; a.mangia@tin.it

Biomarkers can be defined as measurable characteristics to be evaluated as indicators of normal or pathogenic biological processes, or as predictors of treatment response [1]. In the field of viral hepatitis, biomarkers are used to identify patients with HBV-, HDV-, or HCV-related infection, to predict or follow liver disease progression or to pre-identify treatment responses, including responses to experimental agents in clinical trials.

This Special Issue of *Cells* is devoted to various aspects of biomarkers research and application in hepatology, and provides an overview of a number of biomarkers used, for example, to measure the effect of broadening diagnostic interventions (such as the screening for viral infections).

In more detail, the WHO has set ambitious goals for the elimination of hepatitis B and C as a public health threat by 2030 [2]. While birth dose vaccination represents the best option to prevent HBV-related liver disease, access to HBV diagnostic tests varies worldwide for individuals already affected by HBV. As shown by the Polaris Observatory, more than 75% of HBV infections are in low-income countries [3]. Treatment of HBV is cheap, effective, and largely available; therefore, HBV testing strategies need to be implemented and screening campaigns need to be easily accessible.

Resource-constrained areas are less likely to have access to laboratory tests that measure viral levels or detect viral variants. They have a greater reliance on serologic tests. Rapid, cheap, and accurate serological tests are urgently needed in difficult-to-access populations. Increasing testing accuracy and integrating specimen and sample preparation in a simple testing assay is the most challenging aspect of point of care (POC) tests in viral hepatitis elimination strategies. Novel biomarkers that are serologically evaluated may represent a valid alternative for molecular testing, which require trained technicians. In recent years, significant advances have been made to ameliorate and standardize the accuracy of HBsAg POC testing. However, limitations still exist in sample collection, such as the number of required steps and mandatory training, which may compromise screening initiatives. 

In parallel, as the quantification of HBV DNA is fundamental in treatment decisions, a faster quantitation, performable by handheld devices—not as expensive as those currently available—is needed. All these aspects, together with a detailed cost-effectiveness analysis, have been presented and discussed by Xiao Y et al. [4] in this Special Issue of *Cells*.

Finally, as we were left behind in the elimination plans for both HCV and HBV, diagnostic biomarkers identification and POC should also be implemented for HCV. For example, the diagnostic use of HCVAg should be increased in economically disadvantaged countries, and so too should dry-spots kits that can be ordered online and sent directly to the patient for self-assessment [5,6].

Biomarkers can be used in therapeutical decisions. This is the case for the identification of non-cirrhotic HBeAg negative patients for treatment discontinuation after NAs-induced long-term viral suppression and persistent HBsAg positivity. The availability of a virological parameter or set of parameters capable of identifying candidates to NAs discontinuation with no risk of relapse would be necessary. As shown by Papatheodoridis et al., relapse after NAs withdrawal remains highly unpredictable [7]. Asian patients are less likely to benefit from NAs discontinuation, and analysis of given biomarkers cannot be applied regardless of race. Unfortunately, any of the new viral markers as hepatitis B core-related Antigen (HBcrAg) and serum HBV RNA levels have yet been shown to accurately predict relapse after NAs discontinuation.

Moving to the new epidemic of metabolic-associated fatty liver disease (MAFLD), which concerns one in four people worldwide, the identification of new biomarkers to orient risk stratification and therapeutical strategies in NAFLD has become the absolute priority in the field. To overcome risks and limits of histological diagnosis of NAFLD/NASH, imaging tools, able to monitor non invasively fibrosis development and progression in patients with MAFLD, or soluble biomarkers have been evaluated. Many of these biomarkers have been confirmed against liver histology, still representing the gold standard for NAFLD/NASH diagnosis. In this Special Issue, a list, albeit not exhaustive, of these biomarkers has been reported by Piazzolla et al. [8]. Algorithms based on a combination of serum liver function tests, serum lipids evaluations, and biomarkers have been developed, and some were validated against liver biopsy. 

The use of these biomarkers in clinical studies aiming to assess new compound efficacy has been shown to be extremely useful. The prevention of fibrosis progression in NASH is thought to be a likely surrogate endpoint for late-stage liver-related clinical events [9]. Understanding early how investigational products can modify fibrosis progression and the natural history of NASH may be valuable information for developing new therapeutical compounds. For this reason, the research of biomarkers needs to go together with clinical research in this specific field and in the field of hepatocellular carcinoma (HCC).

To investigate biomarkers associated with the risk of HCC in patients with cirrhosis of different etiology, Dr Pinero and colleagues have performed a complete, critical analysis of HCC biomarkers of surveillance, diagnosis, treatment, and prognosis [10]. Of course, as the development of HCC cannot be reliably predicted nowadays, markers of surveillance are the most important to address. Interestingly, the implementation of scores combining demographic and laboratory parameters and capable of providing numerical results and thresholds associated with HCC low, moderate, or high risk, improved HCC prediction [11]. A predictive model combining age, gender, and logarithmic transformation of three biomarkers (AFP, AFP-L3, and DCP) with patients’ characteristics, the GADAL score, has been developed by Professor Johnson [12]. It predicts HCC with an AUROC of 0.97, irrespective of etiology and disease stage. Although based on a very large number of data, the retrospective collection makes it not entirely free from bias. The model significantly increases the detection of early HCC over US alone [0.92 vs. 0.82 *p* < 0.01].

The role of biomarkers in HCC prognosis is linked to the unicity of HCC in comparison to other neoplasia. HCC is a complex disorder with a poor overall prognosis. It presents on a background of chronic liver disease, and the interplay between the underlying liver disease and cancer itself drives treatment decisions and prognosis. These specific features led to the development of scores as ALBI grade that include serum bilirubin and albumin but nor tumor imaging [13].

In terms of measurement of treatment response, at variance with other cancers, biomarkers of HCC response in serum and liver tissue, after treatment, are lacking. VEGF, APF and ANG-2 are prognostic factors, but they do not have any predictive value. Most studies have focused on biomarkers predictive of immunotherapy (ICI) efficacy in patients undergoing ICI. However, the lack of standardized assays to detect PD-L1 expression limits the generalizability of data, as for example, those attained in the Keynote-224 phase II trial, the study on pembrolizumab as second-line therapy. The study showed that a combined positive score (CPS) may be an applicable biomarker of ICI response [14].

In contrast with a large number of studies searching for biomarkers of response to ICI, relatively few studies have investigated biomarkers relating to ICI-associated AEs that would be of increasing clinical importance.

The reported evidence seem to underlie that tools for risk stratification and prediction of treatment response are the most critical to develop and this issue needs to be addressed in future studies.
